# The autoimmune burden in juvenile idiopathic arthritis

**DOI:** 10.1186/s13052-017-0373-9

**Published:** 2017-06-14

**Authors:** Elena Tronconi, Angela Miniaci, Andrea Pession

**Affiliations:** 1 Pediatric Unit, Department of Medical and Surgical Sciences, S. Orsola – Malpighi Hospital, University of Bologna, Bologna, Italy; 2 Sant’Orsola-Malpighi Hospital, Via Massarenti 9, 40138 Bologna, Italy

**Keywords:** Juvenile idiopathic arthritis, Children, Autoimmunity, Autoimmune thyroid disease

## Abstract

**Background:**

Juvenile idiopathic arthritis (JIA) is a chronic inflammatory arthritis of unknown origin which can be considered an autoimmune disease (AD). The aim of this study is to analyse the presence of two or more autoimmune diseases (polyautoimmunity) in patients suffering from JIA and to evaluate the occurrence of ADs in their families.

**Methods:**

Seventy-nine patients diagnosed with JIA aged 0–21 years, admitted to the Paediatric Rheumatology Unit, Sant’Orsola-Malpighi Hospital, Bologna were screened for ADs. Parents were asked about the presence of ADs in the living relatives of first and second degree.

**Results:**

Twelve of 79 patients (15.2%) had at least 1 AD associated with JIA. Eight patients (10.1%) suffered from autoimmune thyroid disease (AITD), three patients had celiac disease, three patients suffered from psoriasis, one from alopecia and 1 from insulin-dependent diabetes mellitus. The average age at diagnosis was 13.2 years and the cumulative incidence of AITD was 36%. Seventy-six families were studied for a total of 438 relatives. The prevalence of ADs was 13%, greater in first-degree relatives (16.7%) than in second-degree ones (11.1%). The most common AD was AITD; there was no difference in JIA’s age of presentation between patients with positive and negative familiarity with ADs (*p* > 0.05).

**Conclusion:**

Children and adolescents with JIA present a high autoimmunity burden, most commonly represented by AITD. Familial autoimmunity is not negligible in patients suffering from JIA (almost 50% of patients have at least one relative with an AD) and it should always be carefully examined.

## Background

Juvenile idiopathic arthritis (JIA) is a chronic inflammatory arthritis of unknown origin. It is a complex disease in which both genetic and environmental factors play an important role; it can be considered an autoimmune disease (AD) for the presence of reactivity towards self-structures like synovia and extra-articular involvement (eyes, skin, kidneys, pericardium).

In children affected by JIA the occurrence of other ADs has been described in case-reports [1–3] or small case-series [4–9]; the majority of studies focused on patients rather than on relatives without analyzing both aspects together.

The primary objective was to evaluate the polyautoimmunity in a cohort of children affected by JIA. The secondary objective was to analyse the presence of ADs in their relatives.

## Methods

Children with a diagnosis of JIA according to the International League of Associations for Rheumatology Classification [10] admitted to the Pediatric Rheumatology Unit of Sant’Orsola-Malpighi Hospital in Bologna were included in the study. Patients with active disease as well as those in clinical remission were included.

Blood tests data were retrospectively analyzed. Each patient was screened yearly for thyroid functionality and autoimmunity: free thyroxine (fT4; normal range 9–17 pg/mL), free triiodothyronine (fT3; normal range 2.5–5.5 pg/mL), thyroid-stimulating hormone (TSH; normal range 0.27–4.20 microIU/mL), antithyroglobulin antibodies (TgA; normal value <115 UI/mL) and antiperoxidase antibodies (TPOA; normal value <34 UI/mL) by electrochemiluminescence Immunoassay. Since the time of diagnosis and then annually thereafter, each patient underwent determination of and anti-tissue transglutaminase (tTG) IgA antibodies. Inflammatory bowel diseases (IBD) were screened through the faecal occult blood test (FOB) and faecal calprotectin in case of suggestive symptoms. Subclinical hypothyroidism was defined as a serum TSH above the defined upper limit of the reference range, with a serum fT4 within the reference range. Overt hypothyroidism was defined as raised TSH together with decreased serum thyroid hormone levels. A diagnosis of AITD is made by the demonstration of an elevated concentration of TgA and/or TPOA in serum [11].

Diagnosis of CD was confirmed by small bowel biopsy in patients with positive antibody profile.

A systematic collection of first and second-degree relatives’ data via a questionnaire was performed. The ADs investigated were AITD, rheumatoid arthritis (RA), ankylosing spondylitis, psoriasis, celiac disease (CD), JIA, insulin-dependent diabetes mellitus (IDDM), vitiligo, alopecia, multiple sclerosis (MS), IBD, systemic lupus erythematosus (SLE), connective tissue disease, scleroderma and Sjögren syndrome. Other ADs were classified in the “other” category.

### Statistical methods

Descriptive statistics were used to describe the population characteristics. Comparison of mean values was performed by the Student’s t-test while that between percentages was done by the chi-square test. Odds ratios were calculated with 95% confidence intervals (CIs) and a *p*-value <0.05 was considered significant. Unadjusted time-to-event analyses were performed using the Kaplan–Meier estimate.

## Results

The study included 79 patients (51 females, median age 10.7 ± 4.7 years, range 2.8–21 years) with a diagnosis of JIA. 72,1% of patients suffered from the oligoarthritis form, 2,5% had a positive polyarticular rheumatoid factor (RF), 13,9% had a negative polyarticular factor, 5,1% had a psoriatic and a systemic onset, while 1,3% was the percentage of enthesitis-related subset. Mean age at onset of joint disease was 5.7 ± 3.9 years (range 0.7–15.7 years). Anti-nucleus antibodies (ANA) were found in 56/79 (70.9%) patients.

**Fig. 1:**
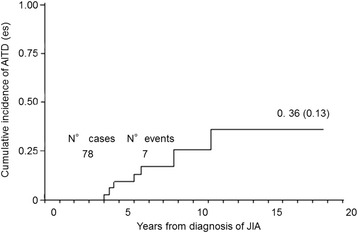
Kaplan Meier estimates of the cumulative incidence of AITD by year of diagnosis of JIA

Eight patients (10.1%) showed AITD. Of these, one was affected by Graves-Basedow disease, 5 had positive TgA and/or TPOA with normal hormones and TSH level, two patients had subclinical hypothyroidism. No correlation between JIA subtype and AITD was noticed (Table 1). The median age at diagnosis of AITD was 13.2 ± 3.1 years and only one patient developed the disease before JIA. Kaplan-Meier estimates of cumulative incidence of AITD were 9.1% at 5 years (*p* = 0.05, CI: 0.03–0.26) and 36% at 18 years after diagnosis (*p* = 0.13, CI: 0.17–0.67) (Fig. 1).

**Table 1:** Clinical and laboratory characteristics in JIA patients with thyroid disorders, coeliac disease, psoriasis, insulin-dependent diabetes mellitus and alopecia

N°	Sex	Age (yrs)	JIA subtype	Hypothyroidism	TgA (UI/mL)	TPOA (UI/mL)	Coeliac disease	IDDM	Psoriasis	Alopecia
1	M	15.2	Psoriasic	−	562	−	−	−	+	−
2	M	16.9	Poli	S	657	−	−	−	−	−
3	F	12.0	Oligo	−	1842	274	−	−	−	−
4	M	11.5	Poli	X	−	−	−	−	−	−
5	F	11.9	Oligo	S	−	−	−	−	−	−
6	F	20.5	Poli	−	448	320	−	−	−	−
7	F	21.0	Oligo	S	256	496	−	−	−	−
8	M	10.9	Sistemic	S	−	−	−	−	−	−
9	F	16.5	Psoriasic	−	−	−	−	−	+	−
10	F	14.1	Oligo	−	178	−	+	−	−	−
11	F	19.1	Oligo	−	129	1300	−	−	−	−
12	F	4.6	Oligo	−	−	−	−	+	−	−
13	F	13.2	Oligo	−	168	138	+	−	−	−
14	M	10.2	Oligo	−	−	−	−	−	−	+
15	F	9.9	Psoriasic	−	−	−	+	−	+	−

Normal range: anti-tyreoglobulin Ab (TgA) < 115 UI/mL, anti-tyreoperoxidase Ab (TPOA) < 34 UI/mL. Hypothyroidism: X present; S subclinic; − absent

CD was present in three patients, all of them were females and affected by another AD (one psoriasis and two AITD). One male patient had alopecia, a female patient had IDDM and three suffered from psoriasis. In conclusion, 12 patients (15.2%) had at least an AD associated with JIA, among these four patients suffered from two ADs (Table 1).

The family history was collected from 76 families because two children were adopted while two were siblings; a total of 438 relatives (53.9% female) were considered. We found that 47.4% of patients had at least one relative with an AD. The prevalence of ADs among relatives was 13%, higher in first-degree relatives (16.7%) compared with second-degree ones (11.1%); 21.6% of mothers suffered from at least one AD compared with 11.8% of fathers (χ^2^ = 1.93, *p* = 0.16); grandfathers were affected in 7.6% of cases while grandmothers in 16.5%(χ^2^ = 2.99, *p = 0.08*). The prevalence of ADs in siblings was 6.7%. The distribution of affected relatives is depicted in Table 2.

**Table 2:** Frequency of autoimmune diseases in the studied families

Number of relatives with history of autoimmune disease	Families
0	40 (52.6%)
1	19 (25%)
2	14 (18.4%)
3	2 (2.6%)
4	1 (1.3%)

Value are number (%) of families, classified by number of parents with history of autoimmune disease. Overall, 47.4% of the families of JIA patients had at least 1 relative with an autoimmune disorder

The most frequent disease was AITD (22 relatives, 95.4% female), both in relatives of first and second-degree (32.8% of all diseases). The second most common disease was psoriasis (20 relatives, 29.8% of all ADs). Other ADs detected were RA (11.9%), vitiligo (6%), alopecia and JIA (4.5%). The presence of the following diseases was also reported: One case of SLE, One systemic vasculitis, One pemphigus, One ulcerative rectocolitis (URC), One Sjögren syndrome and One polyarteritis nodosa (Table 3).

**Table 3:** Prevalence of different autoimmune diseases among relatives of patients with juvenile idiopathic arthritis

Disease	N° of first degree relatives	Sex F %	N° of second degree relatives	Sex F %
AITD	11	100%	11	90.9%
Psoriasis	10	40%	10	60%
Rheumatoid arthritis	3	33,3%	5	80%
Vitiligo	2	50%	2	50%
Alopecia	2	100%	1	100%
JIA	1	100%	2 + 1*	0%
SLE	1	100%	0	
IBD	0		1 URC	100%
Others	1 Pemfigus	0%	1 Sjogren s.	100%
	1 Systemic vasculitis	100%	1 Polyarteritis nodosa	100%

Different disease were divided into first and second-degree relatives. Percentage of female sex individuals for each disease

*+1 refers to one of the siblings suffering from JIA (1 male and 1 female)

Patients with familial autoimmunity were diagnosed with JIA at 5.8 ± 3.9 years while patients with negative family history at 5.2 ± 3.8 years (*p* > 0.05). Seven patients out of 37 with a family history of ADs suffered from two or more ADs compared with 4/40 patients without any family history of this kind of desease (χ^2^ = 0.63, *p* = 0.43).

## Discussion

Fifteen point 2 % of the patients studied had at least one AD in addition to JIA. The thyroid disorders were the most frequent ADs associated (prevalence of 10.1%), generally without alteration in thyroid function, more frequent in the female sex (F:M = 4:1) and not related to a particular JIA subtype. The prevalence of AITD in the pediatric population varies from 1.3% to 9.6% [12]. Graves disease occurs in approximately 0.02% of children (1: 5.000).

CD is an immune-mediated systemic disorder caused by gluten and related-prolamines. Based on a number of studies in Europe and the United States, the prevalence of CD in children between 2.5 and 15 years of age in the general population is 3 to 13 per 1000 children, or approximately 1:300 to 1:80 children [13]. The Guidelines of the European Society for Paediatric Gastroenterogy, Hepatology and Nutrition (ESPGHAN) suggest to test all asymptomatic patients affected by IDDM, Down syndrome, AITD, Turner syndrome, Williams syndrome, IgA deficiency, autoimmune liver disease or with first degree-relatives affected by CD [14]. Although JIA is not included in these guidelines, it is our habit to screen for coeliac antibodies every child with suspected JIA because CD may initially appear with joint involvement. In our population, the 3 patients affected by CD were diagnosed at a mean age of 5.5 years, just before or soon after JIA diagnosis. CD diagnosis was earlier than AITD one (5.5 versus 13.2 years).

In our cohort, we found a prevalence of 10.1% of AITD, 3.8% for CD, 1.3% for IDDM and alopecia. There are few studies on this topic in the literature and the results are often different. Alpigiani et al. studied 66 JIA patients for AITD and IDDM finding a prevalence of 14% and 3%, respectively [4]. Stagi et al. showed a prevalence of 11.9% for AITD and 6.7% for CD [5] while a lower prevalence of AITD was described by Unsal et al., probably because of greater male patients proportion, less affected by thyroid diseases [6]. On the contrary, a higher prevalence was reported by Mihailova et al. [7] and Robazzi et al. [8] (respectively 44.4% and 26%). The heterogeneity of these results may be explained by differences in the populations studied.

There are few case-reports about the association of IDDM and JIA [2, 3] and only one clinical study described this association in 82 patients during a period of 30 years [9].

The incidence of IDDM is highly variable among different ethnic groups; in the United States, the overall prevalence of diabetes among school-age children is approximately 1.9 in 1.000. The annual incidence of new cases in the United States is now approximately 19.7 in 100.000 among youth younger than 10 years and 18.6 in 100.000 of those older than 10 years. Rates are similar or higher in most Western European countries and significantly lower in Asia and Africa [15].

As to alopecia, there are no data about its prevalence in JIA patients and also precise epidemiological information is not available since mild forms often do not reach medical attention. Usually relapsing, the prognosis of alopecia is related to the age of onset, the familiarity, the clinical severity, duration of disease, the presence of other ADs and the response to therapy [16]. In our population, the affected patient developed a total alopecia at the age of 1 year and a half, while the articular disease begun 4 years later. Systemic therapy with Methotrexate has been effective for joint disease and has resulted in a great improvement of the alopecia.

In the literature there is a high number of studies about familiarity for different ADs and these studies were compared in a recent meta-analysis by Cardenas-Roldan [17]. However, there are only few studies about familial autoimmunity in patients affected by JIA. Prahalad et al. [18] underlined how AITD were the most frequent associated diseases while Huang et al. [19] revealed also the presence of psoriasis, ankylosing spondylitis and SLE. Pohjankoski et al. [20] studied the presence of IDDM, CD, MS and chronic arthritis in 355 families of JIA patients showing an overall prevalence of 21.4%. The data of a large cohort of patients in the United States have recently been published [21] showing that the prevalence of autoimmunity is increased among the relatives of JIA subjects compared to those of JDM with a greater proportion of inflammatory arthritis.

Our study confirms the preponderance of autoimmunity in females; this observation can lead to false inference of increased maternal transmission as suggested in previous studies in different diseases [22–24]. However, in the present study, the difference in incidence between the two sexes has not reached statistical significance.

The prevalence of ADs was lower in siblings than in other relatives and this result is probably linked to the young age of subjects.

**Fig. 2:**
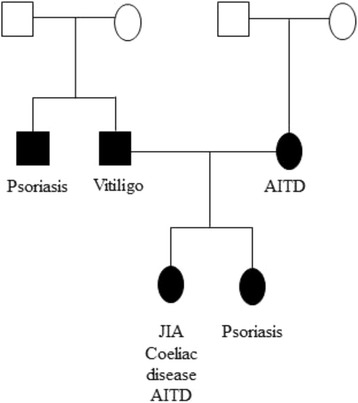
Family tree. Example of poliautoimmunity and familial autoimmunity. The patient is 13 years old and affected by JIA, coeliac disease and AITD; her sister is affected by psoriasis, the father by vitiligo and the mother suffers from AITD

In our population, only one family presented both parents affected by an AD and their child was affected by JIA together with CD and AITD (Fig. 2).

The family of the two siblings is the one with the greater number of affected relatives (*n* = 4). The two patients were both suffering from systemic-onset JIA. Moroldo et al. [25] carried out a study on 183 siblings affected by JIA, finding 11 couples with at least one individual affected by the systemic subtype; only two of these pairs were concordant for this subtype (both affected by systemic-onset JIA). This condition of concordance in our patients is a rare event, but we have to mention that their parents are consanguineous (first-degree cousins).

There was no statistically significant difference in age of onset of JIA among patients with a positive family history and those without familiarity for ADs (*p* = 0.8). Therefore, a positive family history for AD does not seem to entail an earlier development of JIA. Moreover, family history of ADs did not correlate with a higher risk of polyautoimmunity in patients suffering from JIA, in contrast with data showed by Hudson et al. in patients with systemic sclerosis [26].

## Conclusions

Our study confirmed the high incidence risk of new ADs in patients affected by JIA even if we are not able to predict which patient will develop an AD, at what time and what disease. Moreover, the studies published till now refer to a pediatric population without a long-term follow-up. It is not clear how different ADs cluster together but the present study showed how the incidence of AITD, the most frequent AD in general population, increases with time from the diagnosis of JIA, becoming very important after 12 years (cumulative incidence of 36%). Other ADs can develop early, even before arthritis.

Although the environmental factors may influence familial aggregation, the genetic load appears to be a relevant aspect. Familial autoimmunity is an important feature and should always be investigated during family history in patients with arthritis. Our study shows that familiarity for ADs is not negligible in JIA patients, since almost half of them had at least one relative affected by an AD. The presence of positive family history for ADs might be useful to guide the diagnostic pathway towards an autoimmune aetiology. Anyway, a positive familiarity for ADs does not correlate with a particular subtype of JIA and does not seem to lead to an earlier development of arthritis. Our study has some limitations like its retrospective nature and the self-reporting of familial history. However, it is the first study that analyzes both polyautoimmunity and familial aggregation of autoimmunity together in JIA patients.

We will need a more comprehensive study of families with multiple ADs to further clarify the role of shared genomic, transcriptomic and proteomic factors.

## Abbreviations

AD: Autoimmune disease
AITD: Autoimmune thyroid disease
ANA: Anti-nucleus antibodies
CD: Celiac disease
ESPGHAN: European society for paediatric gastroenterogy, hepatology and nutrition
FOB: Faecal occult blood
IBD: Inflammatory bowel diseases
IDDM: Insulin-dependent diabetes mellitus
JIA: Juvenile idiopathic arthritis
MS: Multiple sclerosis
RA: Rheumatoid arthritis
RF: Rheumatoid factor
SLE: Systemic lupus erythematosus
TgA: Antithyroglobulin antibodies
TPO: Antiperoxidase antibodies
tTG: Anti-tissue transglutaminase
URC: Ulcerative rectocolitis
